# An Account of the Preparation and Use of the Phosphorated Soda; Being an Abstract of a Paper on the Subject, Inserted in the Journal De Physique for August, 1788

**Published:** 1788

**Authors:** George Pearson

**Affiliations:** Member of the College of Physicians, Physician to St. George's Hospital, and Lecturer on Physic and Chemistry in London


VI. *An account of the preparation and uſe of the Phoſphorated Soda; being an abſtract of a paper on that ſubject, inſerted in the Journal de Phyſique for Auguſt* 1788, *by* George Pearſon, *M. D. member of the college of phyſicians, phyſician to St. George's hoſpital, and lecturer on phyſic and chemiſtry in London, with conſiderable additions, by the author.*

THE new facts contained in this paper relate either to the chemical properties of this double ſalt, or to its uſe as a purgative.

It appears that M. Lavoiſier united the acid of phoſphorus with the foſſil alkali, but without having ſucceeded in obtaining cryſtals from the combination. This compound, he ſays, was gummy, gluey, and of the conſiſtence of turpentine, &c.

M. Fourcroy confirmed the reſult of M. Lavoiſier's experiment.

M. Sage

M. Sage differed from these two chemists in obtaining non-deliquescent crystals, by combining the acid of phosphorus with the fossil alkali, but the other properties or figure of these crystals he does not describe.

Mr. Klaproth relates that he composed a salt similar to the sal mirabile perlatum of Haupt, or the sal fusibile, with the base of natron of Rouelle, by combining this acid with the above alkali; the figure of which double salt, however, is totally different from the salt I composed of the same substances.

Lastly, Mr. Proust having made a lixivium of the salt for making phosphorus of urine, in order to obtain the fusible or microscomic salt, he procured parallelogramic crystals, which he concludes were composed of an acid analogous to the sedative salt that, united with the soda, forms the sal fusibile with the base of natron, whereas the microscomic salt is principally phosphorated volatile alkali. Professor Bergman adopted this opinion, and admitted this salt, supposed to be analogous to the boracic acid, as a particular acid, and gave it a place under the title of the *Perlate Acid*, in his tables of single elective attractions. M. de Morveau considered this substance in the same light, and called it, in in his dictionary, *Acide Ouretique.* Afterwards this fusible salt, with the base of natron, was decomposed and shewn to consist, not of a peculiar acid, analogous to the sedative salt, but of the phosphoric acid and fossil alkali.

These were the facts already discovered concerning the phosphorated soda, when I made it the subject of investigation. The salt I composed, by the combination of the phosphoric acid with the sal soda, is evidently very different in most of its qualities from that combination made by the above chemists; and also different from the sal fusibile with the base of natron, of Rouelle, Proust, &c. and from the perlate salt of Haupt.

In order that other inquirers may account for the difference in the results of the experiments, I shall relate particularly the manner in which I composed the salt which is the subject of this paper.

1st. The phosphoric acid was procured by dephlogisticating phosphorus by the nitrous acid. Five hundred grains of phosphorus, added, in small quantities, successively, to three or four times that quantity of the nitrous acid, the specific gravity of which was 1.5 and diluted with distilled water, afforded, on evaporation, one ounce and two drachms measure, or about 1100

grains, in weight, of a tranſparent brown fluid, which had the unctuoſity and conſiſtence of oil of vitriol; its ſpecific gravity was 1. 80 to 1. 87. Undoubtedly by this method the phoſphoric acid is obtained in the greateſt degree of purity, but the ſalt prepared with it muſt neceſſarily be very expenſive, and it has been found, that the lixivium of the acid of bones evaporated to a due degree of ſpecific gravity, will anſwer equally well; for then it contains little or no vitriolic ſelenites, and conſequently no glauber's ſalt will be formed and mixed with the phoſphorated ſoda. But attention ſhould always be paid by the manufacturer to the figure of the cryſtals; and if he perceives any of the ſhape of glauber's ſalt, ſuch cryſtals may eaſily be removed.

After procuring the phoſphoric acid in the above way, I diſſolved 1400 grains of cryſtallized ſoda (obtained by decompoſing marine ſalt, with litharge, at Mr. Turner's manufactory) in about 2100 grains of diſtilled water, heated to 140 or 150°. of Fahrenheit's Thermometer, to which ſolution I added, by degrees, 500 grains of the above acid of decompoſed phoſphorus, and the efferveſcence having ceaſed, and the mixture boiled a few minutes, I ſet it to ſtand in a ſhallow low veſſel, in a temperate heat of the air, and thus rhomboidal cryſtals formed at the bottom of the veſſel, the quantity of which was from about 1450 to 1500 grains. After having, by repeated evaporations, obtained this weight of rhomboidal ſalt, a ſediment or liquor remained which would not cryſtallize; this, when dry, weighed from 150 to 200 grains.

From the quantity of ſal ſoda required to form the above weight of double ſalt, the manufacturer will readily calculate the expence of it; for that of the acid of bones is very well known. It is thought proper to make another obſervation in this place, of great conſequence, viz. that great care muſt be taken to uſe pure ſal ſoda, at leaſt, that there be no vegetable alkali mixed with it; for in this caſe there is reaſon to believe, from ſpecimens of it now in the market, that this double ſalt will contain the vegetable alkali, and on that account be rather diſagreeable to the taſte. It is not eaſy to perceive the contamination with the vegetable alkali, if the manufacturer uſes the ſalt from barilla, even in its cryſtallized form; a portion of potaſh being ſo intimately mixed with the foſſil alkali as not to be entirely ſeparable by cryſtallization. We cannot be certain of avoiding the mixture of the vegetable with the fossil alkali, if the barilla alkali be employed. Perhaps the only pure aerated fossil alkali in the market is that prepared by Mr. Turner, in his extremely ingenious process of decomposing sea-salt by litharge. It was with his fossil alkali that I prepared the phosporated soda possessed of the qualities here described. This precaution with regard to the choice of the alkali, used in manufacturing this salt, seems particularly necessary, lest a most agreeable and useful medicine should be lost by the Public, in consequence of want of information, or motives of gain.

If 150 or 200 grains of the phosphoric acid more than the quantity above mentioned (viz. 500 grains) be added to 1400 grains of the sal soda, the only difference in the result will be, that the liquor remaining after the crystallization, is an acid, mucilaginous liquor, which reddens turnsole juice, &c. and with more fossil alkali forms phosphorated soda.

2dly. If, on the contrary, 100 or 200 grains of sal soda more than the above quantity (viz. 1400 grains) be added to the quantity of acid already mentioned (viz. 500 grains) the only difference in the issue of the experiment will be that the fluid remaining after the crystallization is is completed, contains fuperabundant foffil alkali, which will form phofphorated foda, on the addition of more phofphoric acid.

3dly. Diffolve 100 grains of phofphorated foda in an equal quantity of boiling water, and add 5, 10, or 20 grains of acid of decompofed phofphorus as above defcribed, and, on cryftallization, phofphorated foda will be found in an acid liquor which will redden fyrup of violets and turnfole, effervefce with aerated alkali, and fhew no property indicating a chemical union between this acid and the double falt.

4thly. On adding, in different proportions, the fal foda to the phofphorated foda, I did not perceive any chemical union between them, but on cryftallization a mixture of phofphorated foda in rhomboidal cryftals, and of fal foda in differently figured maffes.

Thefe four obfervations appear to be decifive, that the phofphoric acid and fal foda unite together only in *one* proportion, by which the rhomboidal falt here treated of is formed; and that if the perlate falt of Haupt, and fufible falt of Rouelle and Prouft, are compofed of the phofphoric acid and foffil alkali, they cannot unite with a frefh quantity of foffil alkali; or, if they can unite with this alkali, it is an error to affirm, that they confift confift of the acid of phofphorus and foffil alkali. Mr. Klaproth's obfervation cannot be juft, viz. that the phofphoric acid, added to the phofphorated foda, forms a compound which changes fyrup of violets green ; and that phofphoric acid, with excefs of foffil alkali, compofes the fufible falt, with the bafe of natron of Rouelle ; and laftly, that the falt obferved by Mr. Prouft, fuppofed to be analogous to the boracic acid, might be produced by taking away the excefs of foda in the fufible falt of Rouelle, by the addition of vinegar, or phofphoric acid.

The fize of the rhomboidal cryftals is various, according to the quantity of the ingredients, the quantity of water, and the temperature of the atmofphere. The largeft and moft exact rhombs form in warm weather in fuch a quantity of water as holds much of the falt in folution ; for, at this cold feafon (December) the cryftals are fmall and very imperfect. The manufacturer therefore, who wifhes to prepare this falt in the neateft manner, fhould cryftallize, in winter, in the heat of a ftove, of about 90°.

The moft perfect and regular cryftals were about one inch in length, and three-fourths of an inch in breadth. They had fix tetraehdral furfaces of a rhomboidal figure; the angles being meafured as exactly as poſſible with a goniometer*, were 60° and 120°.—the ſolid angles were equally 60° and 120°; ſo that the extremity of the cryſtal preſented a triehdral pyramid, the angles of which were 60°. This double ſalt has no alkaline taſte, altho' it ſometimes changes ſyrup of violets green; its flavor in water and mucilaginous liquids, as in broth and gruel, is that of common ſalt, without the leaſt mixture of any nauſeous or bitter taſte. It effloreſces very ſpeedily in the heat of the hand, or in a dry and warm room; but its cryſtals are permanent in cloſe veſſels, or even in a cool and moiſt air. In its cryſtallized form it contains about $\frac{6}{10}$ its weight of ſolid water; ſo that leſs than half the weight of the deaquated ſalt will produce the ſame purgative effects as above twice its weight when cryſtallized.

From ſix to ten drachms of this rhomboidal ſalt operates as a cathartic, with not only as great mildneſs, but perhaps with leſs irritation than any other purgative. This doſe, in a pint of gruel or broth, without any common ſalt, renders them agreeably ſalt. It ſerves the purpoſe

* An inſtrument, called alſo the anglometer, invented by M. Romé de Liſle.

of of giving the flavor of common salt, and resembles it so much, that many patients have taken this purgative in these liquids without perceiving that they were not flavoured with sea-salt. This quantity of phosphorated soda in half a pint of gruel or beef tea, makes them unpleasantly salt, altho' not nauseous, to most people. Experience has shewn that in many cases, where the stomach was in so irritable a state that any other purgative salts would be immediately rejected by vomiting, or occasion intolerable nausea, the patients could retain, with little or no attending sickness, the phosphorated soda given in dilute solution, as in beef tea, barley water, &c.

It must be remembered, that this salt is very unpleasant exhibited with sugar, or in any distilled waters, *e. g.* mint, peppermint, &c. It is as disagreeable as common salt with any saccharine liquids, or distilled waters. But, like common salt, its taste is agreeable to almost all palates in any insipid, or mucilaginous liquid.

The phosporated soda has been found particularly acceptable to habits that are naturally costive, or rendered so by opium and other medicines, for which state it is very disagreeable to take the purging salts in use ; and yet, the nature of their disorders, as in hectic cases, would not allow allow any other kinds of laxatives. In ſuch caſes from three to ſix drachms in a pint of broth or gruel, in the courſe of a day, has removed their coſtiveneſs, without, at the ſame time, their palates being offended or ſtomach rendered uneaſy.

The phoſphorated ſoda is not ſo purgative as an equal weight of Rochelle ſalt; on ac count of the greater quantity of water which the former contains: nor perhaps is it in moſt conſtitutions quite ſo active as the glauber ſalt, in which there is alſo above $\frac{6}{10}$ ſolid water, but it is found that in doſes of from ſix to ten or, at moſt, twelve drachms, it is generally a pretty conſiderable purgative.

In the preſent ſtate of chemiſtry the phoſphorated ſoda cannot be manufactured at nearly ſo little expence as the glauber ſalt, nor even as the Epſom ſalt; but it is a happy circumſtance for the Public, that it is already prepared at a price not much higher * than the Rochelle ſalt, or ſoluble tartar; and this places an agreeable medicine within the compaſs of moſt patients, to whom palatableneſs is any great object.

* Mr. Willis offers the phoſphorated ſoda for five ſhillings a pound.

 The

The demand for this new ſalt has occaſioned ſeveral manufacturers, beſides Mr. Willis, to prepare it. The ſalt I have ſeen prepared by him, in the ſummer, was well cryſtallized, very neat, and apparently free from any extraneous ſubſtance; but altho' I have entire confidence in his fidelity, and the beſt opinion of his accuracy, yet I cannot help expreſſing a wiſh that he would employ (if he does not do ſo already) the foſſil alkali, obtained by decompoſing ſea-ſalt or glauber ſalt, having, as already explained, reaſon to believe this article is liable to be contaminated by the mixture of the vegetable alkali always in the barilla. The compoſition of this ſalt, however, with the alkali of glauber or ſea-ſalt muſt neceſſarily render it more expenſive.

